# Study on the Thermogravimetric Kinetics of Dehydrated Sewage Sludge Regulated by Cationic Polyacrylamide and Sawdust

**DOI:** 10.3390/polym15102396

**Published:** 2023-05-21

**Authors:** Kai Yang, Jianqi Sun, Hongning Liu, Weichao Yang, Lei Dong

**Affiliations:** 1 College of Mechanical and Electrical Engineering, Shijiazhuang University, Shijiazhuang 065000, China; ysuyangk@163.com (K.Y.);; 2 Shijiazhuang Digital Medical Rehabilitation Technology Innovation Center, Shijiazhuang 065000, China

**Keywords:** sludge, pyrolysis, kinetic, cationic polyacrylamide, sawdust

## Abstract

With the continuous increase in sewage-sludge production worldwide, the pyrolytic disposal of sludge has received great attention. To build knowledge on the kinetics of pyrolysis, first, sludge was regulated using appropriate amounts of cationic polyacrylamide (CPAM) and sawdust to study their enhancing effect on dehydration. Due to the effects of the charge neutralization and skeleton hydrophobicity, a certain dose of CPAM and sawdust reduced the sludge’s moisture content from 80.3% to 65.7%. Next, the pyrolysis characteristics of the dehydrated sludge regulated by CPAM and sawdust were investigated at a heating rate of 10~40 °C/min by using TGA method. The addition of sawdust enhanced the release of volatile substances and reduced the apparent activation energy of the sample. The maximum weight-loss rate decreased with the heating rate, and the DTG curves moved in the direction of high temperature. A model-free method, namely the Starink method, was adopted to calculate the apparent activation energies, which ranged from 135.3 kJ/mol to 174.8 kJ/mol. Combined with the master-plots method, the most appropriate mechanism function ultimately obtained was the nucleation-and-growth model.

## 1. Introduction

China’s urban-sewage-treatment problem has become increasingly severe with the continuous acceleration of urbanization over recent years [1]. Activated sludge and its derivation technology are widely used in urban-sewage treatment due to their low cost and wide adaptability [2]. However, sewage treatment can also create additional solid waste, namely sludge, and most hazardous substances in sewage are enriched in sludge. Unfortunately, initially, sludge has a high moisture content, so its dehydration process is very difficult, leading to astonishing energy consumption. In 2022, the total amount of sludge (with a moisture content of 80%) produced in China exceeded 72 million tons, and it is expected to exceed 90 million tons in three years. In addition, hazardous substances in sludge, such as viruses, bacteria, heavy metals, etc., can various forms of air, water, and soil pollution [3]. The long-term neglect of the sludge problem has created a significant gap between the rates of harmless disposal in China and developed countries. The main conventional sludge-disposal options include landfill, soil utilization, and incineration. In 2021, China officially banned the production of organic fertilizers from sludge, which also means that the range of methods with which sludge can be used in land landfill and soil will become increasingly narrow. Incineration treatments can create secondary pollution problems, which are technically complex and costly [4,5]. As an environmentally benign technology, pyrolysis can recover energy with higher utilization efficiency and lower cost, and has aroused great attention. Pyrolysis technology can not only prevent the formation of toxic organic compounds, but also fix heavy metals into solid residue [6]. Moreover, different products can be obtained by changing the temperature and other process parameters [7,8]. Therefore, pyrolysis technology is suitable as a terminal technology for sludge disposal. 

However, the removal of moisture in the early stage of pyrolysis is the most significant challenge limiting the development of technology. Fortunately, the charge of cationic polyacrylamide (CPAM) (positive charge) is opposite to the charge of sludge colloid (negative charge), which means that they can be electrically neutralized [9]. Furthermore, its long polymer chain can exhibit strong adsorption and bridging functions, so CPAM is widely used as a flocculant for various types of sludge. Three main mechanisms can explain the role of flocculants in sludge disposal. Firstly, some flocculants contain certain hydrophobic groups, which can convert the attached water on the surface of the sludge into free water. The second is the electric neutralization effect, which involves the addition of positively charged polyelectrolytes to reduce the repulsive force between sludge particles. The third is surface adsorption and bridging. Polymer flocculants rely on physical and chemical reactions, such as hydrogen bonding, Van der Waals force, and the coordination bonding force generated by active groups on molecules to improve the sludge-dewatering effect. However, the low content of organic matter in sludge is also worthy of attention. Sawdust is a biomass material with high calorific value, and the addition of sawdust into sewage sludge can form a skeleton construction with many pores and channels [10], which greatly improves the dehydration effect. Moreover, the addition of sawdust also increases the calorific value of sludge. 

Due to the advantages of high reproducibility, sensitivity, and reliability, thermogravimetric analysis (TGA) is suitable for analyzing the pyrolysis characteristics of energetic materials, such as coal [11], municipal solid waste [12], agro-food waste [13], and sewage sludge [14]. Affected by high moisture content, the energy consumption during direct pyrolysis is extremely high. Therefore, many researchers focus on co-pyrolysis with other forms of biomass, such as hazelnut shells and wheat straw. Xu et al. (2017) [15] observed inhibitory and acceleratory interactions in the co-pyrolysis of sludge and hazelnut shells. According to their report, inhibitory effects were observed in the early stage of co-pyrolysis (260~450 °C), while accelerating effects were observed in the later stage (450~900 °C). A synergetic effect in sewage sludge and wheat straw co-pyrolysis was discovered by Wang et al. (2016) [16], and it was strongest when the biomass ratio was 60 wt.%. Pyrolysis kinetic data can provide a theoretical basis for reactor design, so thermokinetic analysis is extremely necessary and important for pyrolysis research [17]. Moreover, kinetic parameters, especially the apparent activation energy and the most appropriate mechanism function, are crucial to understand the pyrolysis process. Therefore, the pyrolysis-based kinetic analysis of sludge has been studied in many reports [18,19,20,21,22,23]. Font et al. (2005) [18] proposed a three-parallel-reactions model to study the pyrolysis and combustion of two kinds of sludge. Scott et al. (2006) [19] described a new algorithm to determine the kinetics of sewage-sludge devolatilization. This algorithm assumes that sludge is a mixture of multiple components, and the decomposition of each component is a single first-order-reaction model. The decomposition temperature of biodegradable and non-biodegradable organic matter and carbonates in sewage sludge was investigated by Barneto et al. (2009) [20], and the influence of oxygen on the thermal degradation process was also studied. Othman et al. (2010) [21] systematically studied the effect of the pyrolysis temperature and residence time on the product yields of gas, oil, and char, and the kinetic parameters were achieved by using TGA. Zhai et al. (2012) [22] proposed a new method for calculating the kinetic parameters using the surface-fitting tool in MATLAB. Soria-Verdugo et al. (2017) [23] employed the distributed-activation-energy-model (DAEM) to obtain the pyrolytic and kinetic parameters of both microalgae and sewage sludge separately. Due to the complexity of the organic matter composition in sludge, the exact mechanism of sewage-sludge pyrolysis is still unclear [24]. In general, the methods used to determine the kinetic parameters in the literature can be roughly divided into two groups: model-free methods and model-fitting methods. The model-free methods can avoid the selection of mechanism models to obtain more accurate activation-energy values, represented by the Kissinger–Akahira–Sunose (KAS) method. Simple model-fitting methods, such as the Coats–Redfern (C–R) method, are not recommended due to their insufficient accuracy, especially for complex organic compounds. In particular, the accuracy of the model-fitting method with a single heating rate has been reported to have significant deviations [25]. Although the model-free methods can easily obtain activation-energy values, the reaction model still needs to be further determined on this basis. One of the most effective methods for determining the activation-energy values of complex organic components is the master-plots method [26]. 

In this research, first, an experimental study was conducted on the dehydration effect of sludge enhanced by adding CPAM and sawdust. Next, the co-pyrolysis process of sewage sludge modulated by CPAM and sawdust was analyzed, and a model-free method was used to determine the apparent activation energy. Lastly, the most appropriate mechanism function was inferred using a master-plots method. 

## 2. Materials and Methods

### 2.1. Materials and Mechanical Dehydration Experiment

The sewage-sludge sample (initial moisture content of 80.3%, wet base) and the sawdust sample used in this work were taken from a municipal wastewater-treatment plant and a furniture-processing factory, respectively, in Qinhuangdao in Hebei province. The cationic polyacrylamide (CPAM) was provided by Beijing KangPuHuiWei Technology Co., Ltd. (Beijing, China). In total, 0, 0.25 g, 0.5 g, 0.75 g, and 1 g of CPAM were added to 50 mL of distilled water and stirred uniformly to obtain mixed solutions of different concentrations. Next, the mixed solution was added to the 200 g of sludge of and stirred continuously until completely dissolved. Subsequently, sawdust with dry basis ratios of 0:1, 1:19, 2:18, 3:17, and 4:16 (2.1 g, 4.5 g, 7.0 g and 10.0 g) was added to the mixed sludge and stirred evenly. Next, the sludge after addition of sawdust and CPAM was packed in an industrial filter cloth and maintained at a pressure of 1 MPa for 20 min to dehydrate it. An appropriate amount of sample was taken, weighed, and placed into a 105 °C drying oven at a constant weight, after which the moisture content of sludge cake was calculated. 

To conduct thermogravimetric experiments on samples, the sewage sludge and sawdust samples were air-dried at 105 °C for over 24 h. Subsequently, the samples were ground and sieved to a particle size of <200 μm. The addition of sawdust can strengthen sludge’s dewatering performance, so sawdust was added to raw sewage sludge at dry-basis mass-weight ratios of 0%, 10%, 20%, and 30%, considering the large density difference. A rotary mixer was used to mix the prepared samples evenly. The proximate analysis of samples was performed based on ASTM D5172-89 standard, and the ultimate analysis was conducted by using a Vario EL cube elemental analyzer (Elementar, Hanau, Germany). Finally, Table 1 presents the proximate analysis and ultimate analysis of the measured sludge and sawdust.

### 2.2. Thermogravimetric Analysis

Pyrolytic characteristics of samples were tested in DTA-60AH (Shimadzu, Kyoto, Japan) in a N_2_ (99.999%) atmosphere with a flow rate of 50 mL/min. About 10 mg of sample was loaded into the alumina crucible in each test. Next, the internal temperature was increased from the ambient temperature to 900 °C at different heating rates of 10~40 °C/min. The TG curves were recorded continuously as a function of temperature, and the DTG curves were obtained by differentiating the TG curves. 

### 2.3. Non-Isothermal Kinetic Model of Pyrolysis

Affected by the complexity of organic matter components in sludge, the non-isothermal kinetic model was used to describe the pyrolysis process. For heterogeneous solid-state reaction, kinetic equation can be described as [27]:

(1) $$\frac{d\alpha }{dT}=\frac{A}{B}\exp(-\frac{E}{RT})f(\alpha )$$

where *T*(K) is the absolute temperature, *E* (J·mol^−1^) and *A* (min^−1^) are the apparent activation energy and the preexponential factor, respectively, *β* (°C·min^−1^) is the heating rate, *R* is the universal gas constant, and the *f*(*α*) is the kinetic integral mechanism function. Generally, *α* is defined as the conversion rate of sample at any time, which can be presented as:

(2) $$\alpha =\frac{m_{0}-m}{m_{0}-m_{\infty }}$$

where *m*_0_, *m*, and *m*_∞_ represent the initial, actual, and final mass of the sample, respectively. The integrated form of *f*(*α*) is generally expressed as:

(3) $$G\left(\alpha \right)=\int_{0}^{\alpha }\frac{d\left(\alpha \right)}{f\left(\alpha \right)}=\frac{A}{\beta }\int_{T_{0}}^{T}\exp\left(-\frac{E}{RT}\right)dT\approx \frac{AE}{\beta R}p\left(u\right)$$

where *u* equals *E*/*RT*, and *p*(*u*) is the integral of *T*. The value of *E* can be obtained through the model-free method. However, this is difficult to solve and, thus, different approximate equations were proposed [28]. 

The kinetic triplet can be used to predict the reaction behavior of materials during thermal degradation [29], which can usually be obtained by model-free methods and model-fitting methods. Model-free method is more widely used, since it can be used to obtain the activation energy without considering the mechanism function. Compared to model-fitting methods with a single heating rate, such as the CR method, the model-free method with multiple heating rates yields more accurate calculation results [25]. Three general model-free methods are FWO, KAS, and Starink, which can be expressed in a general form:

(4) $$\ln\frac{\beta }{T^{C}}=\mathrm{const}-\frac{BE}{RT}$$

where C and B vary depending on the method, as shown in Table 2.

However, different integral approximations can lead to differences in calculation results. According to ICTAC Kinetics committee’s recommendation, using the FWO method without an iterative correction procedure results in poor calculation results for *E* [30]. The Starink method was proven to have the highest accuracy among the three methods [31]. This paper only uses the Starink method for calculation, as using multiple calculation methods with different accuracies is meaningless.

In addition, after obtaining the activation energy, further determination of the pyrolysis mechanism function is also needed. The traditional model-fitting method substitutes all possible mechanism functions into the reaction-kinetics equation to obtain a straight fitting line, and compares it with the experiment data. Next, the mechanism function with the highest degree of correlation is selected as the most appropriate reaction mechanism. However, it is often found that multiple mechanism functions have high degrees of correlation. In order to further determine the mechanism function based on the model-free method, master-plots method was introduced to determine the most appropriate mechanism function. After simple processing of experimental data using the master-plots method, the most appropriate mechanism function was obtained by comparing it with the standard curve [32]. The numerical solution for *u* cannot be obtained. In this paper, the 2nd Luke approximation is used to express:

(5) $$p\left(u\right)=\int_{\infty }^{u}-\exp\left(-u\right)/u^{2}du=\frac{\exp\left(-u\right)}{u}\frac{u+4}{u^{2}+6u+6}$$

After integrating Equation (2) and transforming it, the expression of the master-plots method can be obtained:

(6) $$\frac{G\left(\alpha \right)}{G\left(0.5\right)}=\frac{p\left(u\right)}{p\left(u_{0.5}\right)}$$

Table 3 gives the commonly used solid-phase-pyrolysis mechanism function *f*(*α*) and *G*(*α*). Next, a series of standard model curves was drawn and experimental data were processed on both sides of Equation (6), and the two to were compared determine the most appropriate pyrolysis model.

## 3. Results and Discussion

### 3.1. Effect of CPAM and Sawdust on Sludge-Dewatering Performance

**Figure 1:**
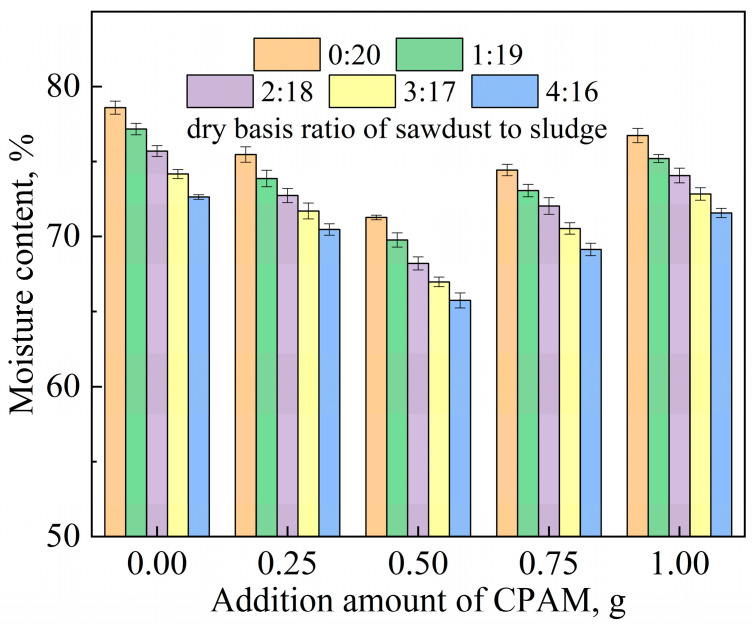
The moisture contents of sludge after mechanical dehydration under different regulatory conditions.

Figure 1 gives the moisture contents of the sludge after mechanical dehydration under different regulatory conditions. When there was no addition, the moisture content of the sludge after the mechanical dehydration was 78.6%. However, it was found that the water content of the dehydrated sludge cake decreased gradually and then increased after adding the CPAM, but all were lower than the case without the addition. This can be explained by the fact that the positive charge carried by the cationic polyacrylamide neutralized the negative charge in the sludge colloid, which changed the structure of the sludge and destroyed the stability of the colloid. The optimal CPAM dosage was 0.5 g, in which case the water content of the dehydrated cake was the lowest (71.5%). The main reason for this is that excessive CPAM introduces redundant positive charge, which makes sludge colloid particles positively charged and, therefore, increases the difficulty of dehydration.

When sawdust was added, the dehydration effect was obviously enhanced compared with that without the addition. The mixing of the sawdust inevitably reduced the water content of the sample to a certain extent, due to its low moisture content. However, the sawdust was only added in small doses as a filter aid, resulting in extremely limited water-content reduction. This suggests that the addition of sawdust can form a skeleton structure, which improves the loose structure of sludge and provides a microporous channel for water to flow out. It is obvious that as the dosage of sawdust increases, the dewatering effect of sludge improves. However, in practice, it is necessary to consider economic factors and daily sludge-treatment capacity, so sawdust should be added appropriately as a conditioner. It can be clearly seen that the superposition of the two effects significantly improves the sludge dewatering performance with the addition of sawdust and CPAM simultaneously. The optimal addition amounts of CPAM and sludge were 10 g and 0.5 g, respectively, causing a decrease in the moisture content of the sludge to 65.7% after mechanical filtration. In conclusion, the use of CPAM and sawdust as conditioners greatly improved the dewatering efficiency of the sludge in two respects: charge neutralization and the construction of hydrophobic channels. From another perspective, the addition of distilled water in this experiment does not necessarily mean that for every ton of sludge, 250 L of water is polluted. In practical applications, pre-sludge filtrate is used instead of distilled water to reduce water pollution.

### 3.2. TG-DTG Pyrolysis Analysis

The addition of sawdust not only contributes to the mechanical wetting of wet sewage sludge as the skeleton construct [10], but also has a significant impact on the coupling pyrolysis process of sludge and sawdust. The density of sawdust is much lower than that of dry sludge, so the amount of sawdust added should be considered in the performance of sewage-sludge dewatering. Considering the economical efficiency and sludge-treatment capability, three dry-basis mass ratios (9:1, 8:2, and 7:3) of sewage sludge to sawdust were chosen to investigate the influence of the sawdust on the pyrolysis characteristics. In addition, considering the extremely small amount of CPAM added, its impact on pyrolysis was not considered.

**Figure 2:**
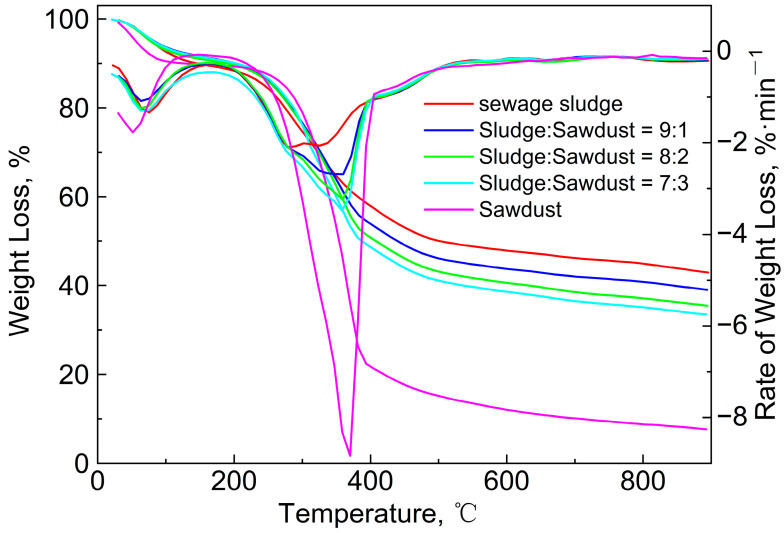
TG/DTG curves of sewage sludge, sawdust, and their blends at the heating rate of 10 °C/min.

Figure 2 shows the TG/DTG curves of the sludge, the sawdust, and their blends at the heating rate of 10 °C/min. Two obvious weight-loss peaks can be clearly observed in DTG curves of the sludge and sawdust. The first peak was caused by the precipitation of adsorbed water and bound water in the samples [12,14,15]. A second peak can be seen within the range of 230 °C to 500 °C with a maximum of around 313 °C for the sewage-sludge sample. Similarly, the main pyrolysis range of the sawdust was approximately 240 to 430 °C, with a maximum of around 370 °C. The weight loss (65%) and the maximum weight-loss rate (8.8%/min) of the sawdust were much larger than those (40% and 2.1%/min) of the sewage sludge. This is mainly attributable to the higher content of volatile and organic phases in sawdust compared to sludge, and the lower ash content of sludge compared to sewage. These factors also led to the differences in the final residual solids corresponding to the TG curves. The main components of sawdust are lignin, hemicellulose, and cellulose, and their decomposition-temperature ranges are 410~540 °C, 198~398 °C, and 300~350 °C [33], respectively, all of which are essentially consistent with the experimental TG curves. The main organic components of sludge are protein, fat, and polysaccharide compounds [14,15,26], and their corresponding decomposition ranges are 300~400 °C, 250~350 °C, and 250~380 °C [34], respectively. The chemical bonds between these compounds are very weak, and when they reach their respective chemical bond breaking or group-transition temperature, two overlapping weight-loss peaks may be found. Therefore, it can be seen that there were two peaks and one valley in the temperature range of 230~500 °C. Finally, the sludge and sawdust experienced a relatively slow weight-loss stage, with weight losses of 18% and 8%, respectively. The mass loss of the sawdust was due to the decomposition of lignin, cellulose, hemicellulose, and other inorganic compounds [35]. The final weight loss may have been caused by the decomposition of residual organics, minerals, metal salts, and other inorganic compounds in the sludge [14].

As shown in Figure 2, the TG/DTG curves of the blends shifted from the sewage sludge to the sawdust as the mass ratio increased, and the peak temperature increased from 313.7 °C to 360.1 °C. Furthermore, the solid residue was reduced and the maximum weight-loss rate increased from 2.1%/min to 3.3%/min. This phenomenon was similar to that observed in studies of the co-pyrolysis characteristics of coal with corn and sugarcane residues [36], low-rank Malaysian coal, oil-palm biomass [37], paper sludge, and municipal solid waste [12]. To evaluate the pyrolysis characteristics of the sludge, sawdust, and their blends, the following pyrolysis characteristic coefficients were introduced:

(7) $$D=\frac{{\left(dw/dt\right)}_{\max}\times {\left(dw/dt\right)}_{ave}}{T_{\max}\times T_{i}\times \Delta T_{1/2}}$$

where (*dw*/*dt*)_max_ (%/min) and (*dw*/*dt*)_mean_ (%/min) represent the maximum and average weight-loss rate, respectively, *T*_max_ (°C) is the corresponding temperature of (*dw*/*dt*)_max_, *T*_i_ (°C) is the temperature at which the volatiles began to release and Δ*T*_1/2_ represents the temperature range of the (*dw*/*dt*)/(*dw*/*dt*)_max_ = 0.5 (half-peak width). Table 4 gives the calculation results obtained by Equation (7).

As shown in Table 4, with the increase in the sawdust ratio, *T*_i_, (*dw*/*dt*)_max_ and (*dw*/*dt*)_mean_ increased, and Δ*T*_1/2_ was obviously narrowed. The *D* value significantly increased from 1.133 to 2.498 as the proportion of sawdust increased. Meanwhile, the (*dw*/*dt*)_max_ and (*dw*/*dt*)_mean_ also increased accordingly, which means that the release of volatile matter became more intense with the increase in the sawdust ratio. The gradually increasing *T*_max_ and *T*_i_ indicate that the weight-loss peak was delayed as the amount of sawdust added increased. However, a decrease in the Δ*T*_1/2_ value indicates a more concentrated peak of mass loss. The changes in these indicators also mean that the addition of sawdust caused a lag in the pyrolysis-reaction temperature and an increase in the reaction rate.

### 3.3. Effect of the Heating Rate on Pyrolysis Characteristics

**Figure 3:**
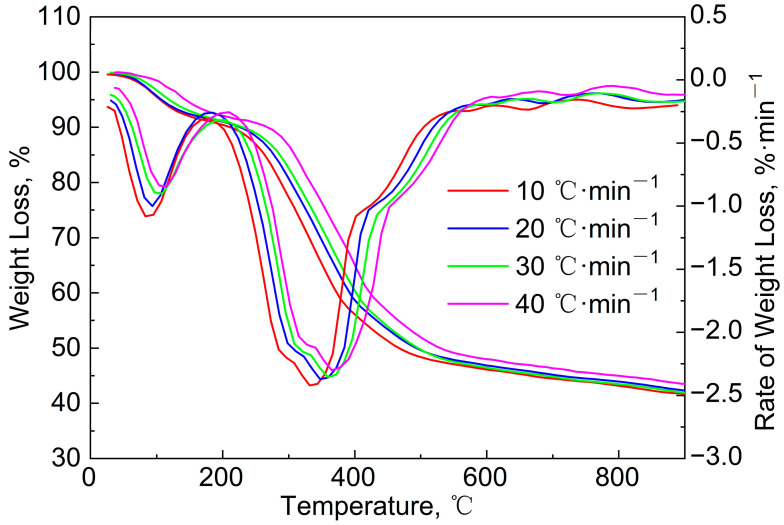
TG/DTG curves of blends (mass ratio = 9:1) at different heating rates.

Figure 3 gives the TG/DTG curves of the blends (mass ratio = 9:1) to study the effect of the heating rate (*β*) on the pyrolysis process. It can be seen that these TG curves are similar, but the (*dw*/*dt*)_max_ decreased from 2.6%/min to 2.2%/min with the increase in *β*, and the quality of the solid residues increased. This is because the faster heating rate led to the shortening of the time required for the pyrolysis reaction, and the non-thermal-equilibrium state occurred inside the sample particle. Gaseous products may not have been released in time, which would have prevented the pyrolysis reaction from proceeding smoothly. In addition, due to the heat-transfer effect inside the sludge particles, a faster heating rate would have resulted in a larger temperature gradient inside the particles, which would also have affected the progress of the internal pyrolysis reactions. Therefore, the sample pyrolysis was not complete when the temperature reached 900 °C, and the solid residue increased. Furthermore, the DTG curves and the value of *T*_max_ moved in the high-temperature direction. However, the movement trend was not obvious. The differences between the *β* of 20, 3,0 and 40 °C/min are small, but the DTG curve at 10 °C/min was much lower than the other three curves. This phenomenon was similar to those of the DTG curves found in the literature [24].

### 3.4. Thermal Kinetics Analysis

**Figure 4:**
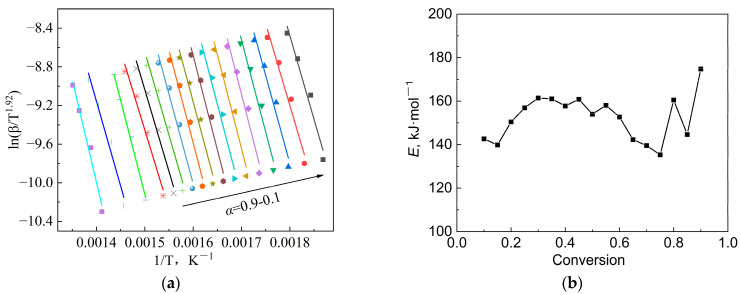
Regression results of the experimental data: (**a**) Starink method and (**b**) *E* value at different conversion rates using different methods.

The magnitude of the apparent activation energy determines the pyrolytic reaction rate, which is the minimum energy required for the initiation of a pyrolytic reaction. As mentioned above, a model-free method named Starink was chosen to calculate *E*, as its accuracy is higher than that of the KAS and FWO methods. Figure 4a gives the regression diagram of the Starink method with the conversion rate *α* of 0.1~0.9, and Figure 4b shows the variation in the *E* value with *α* calculated by different methods. It can be seen that the correlation between the results from the three methods was quite strong. The activation-energy values calculated by the Starink method ranged from 135.3 kJ/mol to 174.8 kJ/mol. It is worth noting that this result was lower than the calculation results using the FWO method. Similar results were found in the literature [38]. The *E* value gradually increased with the increase in *α* in the early stage, but it actually decreased when the conversion rate exceeded 0.4. In the final stage, *E* showed a significant increase again when *α* exceeded 0.7. This can be attributed to the complexity of the pyrolytic reaction. The complex composition of the blend results in multi-step reactions during pyrolysis, such as continuous reactions, competitive reactions or parallel reactions [25]. Therefore, this phenomenon is almost inevitable during the pyrolysis of highly complex organic matter, such as sludge. In the early stage of pyrolysis, some types of organic component begin to decompose, resulting in a slight increase in the apparent activation energy and a decrease in the reaction rate. After reaching a certain temperature range, the decomposition of these components is completed successively, while other organic components begin to decompose one after another. After another period of time, the main organic matter gradually decomposes, and the reaction rate gradually decreases. The occurrence of this series of reactions results in significant fluctuations in activation energy during the pyrolysis process. However, it is difficult to determine whether these multi-step reactions are independent, competitive, reversible, or continuous. Here, we only treat this series of reactions as a whole, from a macro perspective.

**Figure 5:**
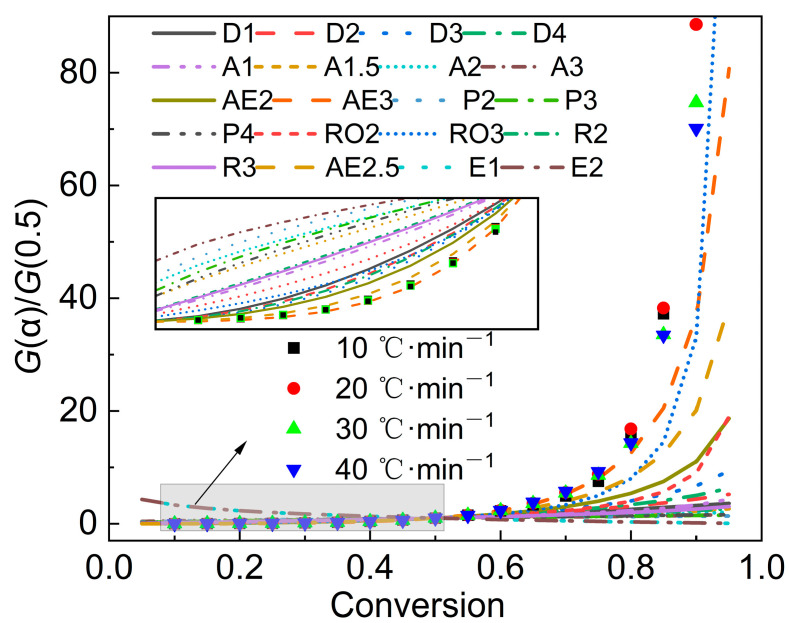
Calculation results of the master-plots method.

Furthermore, the most appropriate reaction function can be evaluated by the master-plots method. By substituting the conversion rate into the function in Table 3 to obtain a series of *G*(*α*), and then drawing *G*(*α*)/*G*(0.5) versus *α*, a series of standard curves can be formed. By comparing the experimental data with the standard curves, the most appropriate mechanism function can be determined. Figure 5 gives the calculation results of the master-plots method. Due to the complexity of the sludge’s composition, the experimental data did not coincide with any of the standard curves. When *α* was lower than 0.5, the curve AE3 was more consistent with the experimental data points, indicating that the pyrolytic mechanism belonged to the nucleation-and-growth model. However, in the later stage, as the *α* increased, there were some deviations between the AE3 model and the experimental data, especially when the *α* exceeded 0.8, which may be attributed to the large fluctuation in the *E* value and the complex decomposition reactions in the later stage of pyrolysis. However, in general, it can be considered that the most probable mechanism function in the main pyrolysis stage is the nucleation-and-growth model.

## 4. Conclusions

In this work, sewage sludge was regulated using CPAM and sawdust to study their enhancing effect on dehydration, and then the pyrolytic kinetics of dehydrated sludge regulated by CPAM and sawdust were established in order to determine the pyrolytic mechanism. The use of CPAM and sawdust as conditioners greatly improved the dewatering efficiency of the sludge in two respects: charge neutralization and the construction of hydrophobic channels. The optimal amounts of added CPAM and sludge were 10 g and 0.5 g, respectively, resulting in moisture contents of sludge after mechanical dehydration ranging from 80.3% to 65.7%. The maximum weight-loss rate of the sawdust was four times higher than that of the sludge. With the increase in the sawdust ratio, the release of volatile matter became more intense, and the apparent activation energy reduced in the main pyrolysis stage. Non-isothermal TGA data were analyzed by using a model-free method and the master-plots method to obtain the apparent activation energy and the most appropriate mechanism function. The apparent activation energy was calculated using a model-free method, namely the Starink method, with a range of 135.3 kJ/mol to 174.8 kJ/mol. The most appropriate mechanism function ultimately obtained was the nucleation-and-growth model. The results of this research can provide theoretical support for the dehydration process and the design of pyrolysis reactors. However, the effect of the heat-transfer efficiency on the pyrolysis process of large-particle sludge in actual pyrolytic processes is worth considering and studying.

## Figures and Tables

**Table 1:** Proximate analysis and ultimate analysis of samples.

Proximate Analysis (As-Received Basis)		
	Sludge	Sawdust
Moisture, %	5.21	3.05
Volatile Matter, %	60.38	78.62
Fixed Carbon, %	4.96	17.01
Ash, %	29.45	1.32
Ultimate Analysis (Dry Basis)		
C, %	23.98	48.62
H, %	4.26	6.81
N, %	4.01	0.32
S, %	1.58	0.11
O, % (by difference)	19.08	43.35
LHV (kJ/kg)	12,182	18,325

**Table 2:** Values of C and B for three general model-free methods.

Method	C	B
KAS	2	1
FWO	0	1.052
Starink	1.92	1.008

**Table 3:** Commonly used solid-phase-pyrolysis mechanism function.

Model	*f*(*α*)	*G*(*α*)	Model	*f*(*α*)	*G*(*α*)
A1	1 − *α*	−ln(1 − *α*)	D1	1/2*α*^−1^	*α* ^2^
A1.5	3/2(1 − *α*)[−ln(1 − *α*)]^1/3^	[−ln(1 − *α*)]^2/3^	D2	[−ln(1 − *α*)]^−1^	*α* + (1 − *α*)ln(1 − *α*)
A2	2(1 − *α*)[−ln(1 − *α*)]^1/2^	[−ln(1 − *α*)]^1/2^	D3	3/2(1 − *α*)^2/3^[1 − (1 − *α*)^1/3^]^−1^	[1 − (1 − *α*)^1/3^]^2^
A3	3(1 − *α*)[−ln(1 − *α*)]^2/3^	[−ln(1 − *α*)]^1/3^	D4	3/2[(1 − *α*)^−1/3^ − 1]^−1^	1 − 2/3*α*-(1 − *α*)^2/3^
AE2	1/2(1 − *α*)[−ln(1 − *α*)]^−1^	[−ln(1 − *α*)]^2^	RO2	(1 − *α*)^2^	(1 − *α*)^−1^ − 1
AE2.5	2/5(1 − *α*)[−ln(1 − *α*)]^−1.5^	[−ln(1 − *α*)]^2.5^	RO3	(1 − *α*)^3^	−1/2[1 − (1 − *α*)^−2^]
AE3	1/3(1 − *α*)[−ln(1 − *α*)]^−2^	[−ln(1 − *α*)]^3^	R2	2(1 − *α*)^1/2^	1 − (1 − *α*)^1/2^
P2	*α* ^1/2^	2*α*^1/2^	R3	3(1 − *α*)^2/3^	1 − (1 − *α*)^1/3^
P3	*α* ^1/3^	3*α*^2/3^	E1	*α*	ln *α*
P4	*α* ^1/4^	4*α*^3/4^	E2	1/2*α*	ln *α*^2^

**Table 4:** Pyrolysis-characteristics parameters of samples at different ratios.

Mixing Ratio	10:0	9:1	8:2	7:3
(*dw*/*dt*)_max_/%·min^−1^	2.1	2.6	3.2	3.4
(*dw*/*dt*)_mean_/%·min^−1^	0.64	0.69	0.73	0.74
*T*_max_/°C	313.7	358.7	359.4	360.7
*T*_i_/°C	220.2	226.1	230.3	232.3
Δ*T*_1/2_/°C	171.7	126.5	124.7	120.2
*D*/10^−7^%^2^·min^−2^·°C^−3^	1.133	1.748	2.263	2.498

## Data Availability

The data presented in this study are available on request from the corresponding author.
